# The "etiome": identification and clustering of human disease etiological factors

**DOI:** 10.1186/1471-2105-10-S2-S14

**Published:** 2009-02-05

**Authors:** Yueyi I Liu, Paul H Wise, Atul J Butte

**Affiliations:** 1 Stanford Medical Informatics, Department of Medicine, Stanford University School of Medicine, Stanford, CA, USA; 2 Department of Pediatrics, Stanford University School of Medicine, Stanford, CA, USA; 3 Lucile Packard Children's Hospital, Palo Alto, CA, USA

## Abstract

**Background:**

Both genetic and environmental factors contribute to human diseases. Most common diseases are influenced by a large number of genetic and environmental factors, most of which individually have only a modest effect on the disease. Though genetic contributions are relatively well characterized for some monogenetic diseases, there has been no effort at curating the extensive list of environmental etiological factors.

**Results:**

From a comprehensive search of the MeSH annotation of MEDLINE articles, we identified 3,342 environmental etiological factors associated with 3,159 diseases. We also identified 1,100 genes associated with 1,034 complex diseases from the NIH Genetic Association Database (GAD), a database of genetic association studies. 863 diseases have both genetic and environmental etiological factors available. Integrating genetic and environmental factors results in the "etiome", which we define as the comprehensive compendium of disease etiology. Clustering of environmental factors may alert clinicians of the risks of added exposures, or synergy in interventions to alter these factors. Clustering of both genetic and environmental etiological factors puts genes in the context of environment in a quantitative manner.

**Conclusion:**

In this paper, we obtained a comprehensive list of associations between disease and environmental factors using MeSH annotation of MEDLINE articles. It serves as a summary of current knowledge between etiological factors and diseases. By combining the environmental etiological factors and genetic factors from GAD, we computed the "etiome" profile for 863 diseases. Comparing diseases across these profiles may have utility for clinical medicine, basic science research, and population-based science.

## Background

Etiologies of human diseases include both genetic and non-genetic (here considered as "environmental" in the broadest sense) factors, such as behaviors and chemical exposures. Here, we are defining disease broadly, including syndromes, accidents, and conditions. An example of a disease whose incidence is influenced primarily by genetic factors is cystic fibrosis, an autosomal recessive disease from a mutation in the cystic fibrosis transmembrane conductance regulator (CFTR). On the other end of the spectrum, mesothelioma is a disease that is almost always a result of previous exposure to asbestos. The vast majority of diseases, however, are influenced by a large number of both genetic and environmental factors. In a recent analysis of Scandinavian twin registries, genetic factors were estimated to account for 27% of the risk factors for breast cancer, 35% for colon cancer, and 42% for breast cancer, with the remaining percentages due to environmental factors [1]. In addition, genetic and environmental factors almost never act independently. A classical example is Phenylketonuria (PKU): even with the genetic mutation, the disease can be clinically avoided by eliminating phenylalanine from the diet.

Decades-long efforts to sequence the human genome have yielded substantial tools to comprehensively survey positions of DNA variation [2]. These tools have been used to study genome-wide association and linkage between variations and diseases successfully yielding an impressive list of genes associated with diseases. Though most studies focus on either a single gene, loci, or a single disease, many databases, such as Online Mendelian Inheritance of Men (OMIM) [3], PharmGKB [4], and the NIH Genetic Association Database (GAD) [5], have been created to collect such disease-gene associations.

The environmental contributions to diseases have also been widely studied. Similar to genetic associations, most studies focus on singular diseases. So far, to our knowledge, there has been no comprehensive list of environmental factors and their disease associations, similar to the databases listed above. This lack of a comprehensive list is likely due to the fact that environmental factors are a heterogeneous group spanning many categories, and even representing these factors is a challenge.

In this paper, we present a first-step towards the human disease "etiome," which we define as the comprehensive compendium of both genetic and environmental etiological factors associated with diseases. The disease-gene associations are obtained from the Genetic Association Database (GAD). The disease-environmental factor associations are obtained from a comprehensive analysis of the MeSH (Medical Subject Heading) annotations of published literature in the MEDLINE database. After integrating to create a single master set of genetic and environmental etiology factors and diseases, we cluster the etiological factors and determine how genes and environmental factors share similar disease profiles.

## Results

We first sought to identify and integrate lists of genetic and environmental etiological factors associated with diseases. Our search of the Genetic Association Database (GAD) [5] resulted in 5,727 disease-gene associations between 1,034 diseases and 1,100 genes. Table 1 lists the top 10 genes with variants associated with the greatest number of diseases. Angiotensin converting enzyme (*ACE*) has been associated with more distinct diseases and conditions than any other human gene.

**Table 1:** Top 10 genes having variants associated with the greatest number of diseases, as identified from the NIH Genetic Association Database.

**Gene Symbol and Name**	**Count of Distinct Associated Diseases**
ACE (angiotensin I converting enzyme)	100
TNF (tumor necrosis factor alpha)	88
TP53 (tumor protein 53)	73
VDR (vitamin D receptor)	66
HLA.DRB1	59
APOE (apolipoprotein E)	56
NOS3 (nitric oxide synthase 3)	54
MTHRF (methylenetetrahydrofolate redutase)	53
HLA.DQB1	46
IL10 (interleukin 10)	46

Literally hundreds of thousands of articles have been published on the environmental contributions to individual diseases. Representing these environmental factors and searching the literature for these articles describing disease-environmental factor relationships would be a daunting task if it were not for Medical Subject Headings (MeSH), a controlled vocabulary created by the National Library of Medicine (NLM) to index journal articles and books in the life sciences. All articles in MEDLINE have been annotated with MeSH by NLM curators or designees. The 2007 version of MeSH has 24,357 subject headings organized in 16 categories including diseases, drugs and chemicals, and anatomical terms. MeSH also uses 83 qualifiers, which can be added to subject headings to emphasize a particular aspect of the headings. For example, *Lung Neoplasms *is a subject heading and *etiology *is a qualifier; *Lung Neoplasms/etiology *describes the subheading of articles on the etiology of lung neoplasms. Every article in MEDLINE is indexed with 10–15 subject headings, a few of which are designated as *major *(i.e., key points of the article), often designated with an asterisk. MeSH annotation of MEDLINE provides a knowledge source that is concise and amenable to computational approaches.

We downloaded MeSH annotations of all articles in MEDLINE (over 4.6 million articles) that were published between 1996 and 2006 from the MEDLINE Baseline Repository . We examined the definition and purpose of MeSH qualifiers as published in the MeSH Indexing Manual (Chapter 19 of the Indexing Manual, ) to identify qualifiers that encode etiological information for diseases. We found the appropriate qualifiers include *chemically induced*, *complication*, *adverse effects*, *toxicity*, and *poisoning*. These qualifiers, together with their scope note from Chapter 19 of the Indexing Manual and sample indexing, are listed in Table 2. Only those subject heading/qualifier annotations that are designated as major are examined. Note that the qualifier *complication *can be used in two ways. First, the qualifier may annotate one disease causing another. Second, the qualifier or the co-existence of two or more diseases for which no causal relationship has been determined. These two scenarios can be differentiated by whether one of the diseases is annotated with *etiology*. In these cases, our study only included articles annotated as one disease causing another.

**Table 2:** Scope and sample indexing of the MeSH qualifiers used in our study. The five MeSH qualifiers relevant to this study are listed, from the 83 available. Scope notes are taken from the Chapter 19 of the MeSH Indexing Manual .

**MeSH Qualifier**	**Scope Note**	**Example indexing** **(MeSH subject Heading/qualifier)**
Chemically induced (chem ind)	"Used for biological phenomena, diseases, syndromes, congenital abnormalities, or symptoms caused by endogenous or exogenous substances."	Indomethacin-induced peptic ulcer: Peptic Ulcer/chem ind Indomethacin/adv eff

Complication (compl)	"Used with diseases to indicate conditions that co-exist or follow, i.e., co-existing diseases, complications, or sequelae."	If disease A causes disease B, this will be indexed as Disease A/compl Disease B/etiol If it is not known whether disease A causes disease B or disease B causes disease A, the article will be indexed as Disease A/compl Disease B/compl

Adverse Effects (adv eff)	"Used with drugs, chemicals, or biological agents in accepted dosage – or with physical agents or manufactured products in normal usage – when intended for diagnostic, therapeutic, prophylactic, or anesthetic purposes. It is used also for adverse effects or complications of diagnostic, therapeutic, prophylactic, anesthetic, surgical, or other procedures, but excludes contraindications for which "contraindications" is used."	Hepatotoxicity of acetaminophen given for fever: Acetaminophen/adv eff/ther use Liver Diseases/chem ind Fever/drug ther

Toxicity (tox)	"Used with drugs and chemicals for experimental human and animal studies of their ill effects. It includes studies to determine the margin of safety or the reactions accompanying administration at various dose levels. It is used also for exposure to environmental agents. Poisoning should be considered for life-threatening exposure to environmental agents."	Cocaine induced cardiomyopathies: Cocaine/tox Cardiomyopathies/chem ind

Poisoning (pois)	"Used with drugs, chemicals, and industrial materials for human or animal poisoning, acute or chronic, whether the poisoning is accidental, occupational, suicidal, by medication error, or by environmental exposure."	Acidosis due to ethylene glycol poisoning: Acidosis/chem. ind Ethylene Glycol/pois

Our search of the MeSH annotations from the MEDLINE database resulted in 133,781 disease-environmental factor relationships between 3,159 diseases and 3,342 environmental factors. To capture the various categories that these environmental factors belong to, we grouped them based on their semantic types in the Unified Medical Language System (UMLS). The environmental factors belong to 78 distinct semantic types. Table 3 lists the top 10 semantic types of the environmental factors and the number of factors belonging to each type. The top category is *Disease or Syndrome*, reflecting disease progression or complications of a disease. An example of this is the disease *diabetes *leading to the disease *diabetic neuropathy*. Beyond this, thousands of drugs and chemicals have been linked etiologically to disease. Table 4 lists the ten etiological factors that are involved in the greatest number of diseases.

**Table 3:** Top 10 semantic types of the environmental factors causing disease, as represented in the Unified Medical Language System (UMLS).

**Semantic Type of Environmental Factors**	**Count**
Disease or Syndrome	1932
Pharmacologic Substance	1250
Organic Chemical	1076
Therapeutic or Preventive Procedure	521
Neoplastic Process	424
Amino Acid, Peptide, or Protein	310
Biologically Active Substance	274
Hazardous or Poisonous Substance	251
Congenital Abnormality	160
Injury or Poisoning	150

**Table 4:** Top ten etiological factors involved with the greatest number of diseases

**Type of Etiological Factors**	**Count of Distinct Associated Diseases**
HIV infections	203
Lupus Erythematosus, Systemic	181
Smoking	171
Antineoplastic Agents	167
Bone Marrow Transplantation	158
Immunosuppressive Agents	156
Renal Dialysis	154
Kidney Transplantation	151
Antineoplastic Combined Chemotherapy Protocols	138
Acquired Immunodeficiency Syndrome	128

Even the top ten factors span a number of semantic types. The role of Systemic Lupus Erythematosus (SLE) and HIV in leading to successive conditions is well known. The incidence of HIV infection in the population is at least an order of magnitude higher than the incidence of SLE; that both of these make up the top two factors suggests that our relations are not necessarily biased by incidence. Most of the top ten etiological factors are medical procedures themselves, and may reflect the continual shift of disease management from acute and semi-acute management of a life-threatening condition to the chronic management of subsequent complications. Smoking is the only human behavior listed in the top ten.

The disease-environmental relationship with the greatest number of articles is *heparin *with *thrombocytopenia *(508 articles). Other associations with over 300 articles include *carcinogens *with *neoplasms*, *environmental exposure *with *occupational disease*, and *hormone replacement therapy *with *breast neoplasms*.

### The human disease "etiome"

After integrating relationships extracted from GAD and MEDLINE, we found 863 diseases have associations with both genetic and environmental factors. Combining both types of etiological factors for these diseases resulted in 69,223 associations between 863 diseases and 4173 etiological factors.

**Figure 1:**
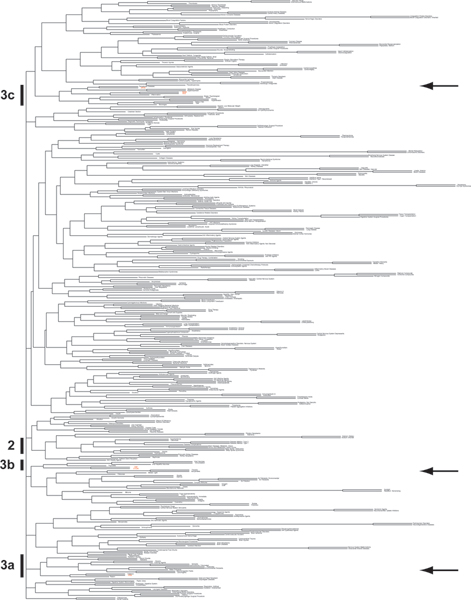
**Clustering of genetic and environmental factors together, based on similarity of profiles of diseases for which they are etiological**. Only the top 427 etiological factors based on counts of articles are shown arbitrarily, for visibility. Arrows indicate the position of genes across this clustering, showing that genes do not all cluster together. Numbers and letters on the left indicate regions magnified for subsequent figures. Please see the journal's website or our website  for a PDF version of this figure that can be magnified.

We clustered all the etiological factors (both genetic and environmental) based on similarities of their disease associations (Figure 1; for details, see Methods). The strongest pairings of environmental factors include some pairings that could be considered "is a" relationships, such as *Parkinsonian Disorders *with *Neurodegenerative Diseases*, *Sunlight *with *Nonionizing Radiation*, and *Tissue Transplantation *with *Organ Transplantation*, as well as many non-"is a" relationships, like *Anterior Uveitis *with *Mouth Diseases*, *Mental Retardation *with *Chromosome Disorders*, and *Platinum Compounds *with *Chlorine Compounds*.

**Figure 2:**
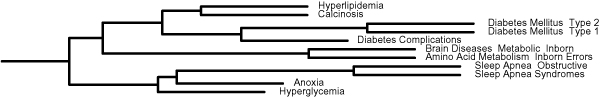
**The diabetes cluster**. As etiological factors, type 1 diabetes mellitus and type 2 diabetes mellitus share similar disease profiles with calcinosis, hyperlipidemia, sleep apnea, and inborn errors of metabolism. Branches are shown shorter here and in Figure 3 as compared to Figure 1, to improve readability of text. The branching patterns and relative lengths of branches are identical to Figure 1.

Overall, etiological factors affecting the same organ systems tend to cluster together. One example is a cluster that has diabetes mellitus type 1, diabetes type 2, and hyperglycemia (Figure 2). As diseases, type 1 and type 2 diabetes have distinctly different etiology. Type 1 diabetes results from the destruction of beta cells within the islets in the pancreas, whereas type 2 diabetes results from initial insulin resistance. However, they lead to the same set of complications including accelerated atherosclerosis, neuropathy, retinopathy, nephropathy, and infections. It is this shared profile of complications that makes diabetes type 1 and type 2 cluster next to each other. This example also illustrates how diseases can also be etiological factors for other diseases. Other examples (data not shown) include a cluster of anticoagulants such as heparin and Coumadin, a cluster of infections from CMV, RSV, gram negative bacteria, and streptococcus, and a cluster that suppress the immune system such as heart transplantation, lung transplantation, and immunosuppression.

However, some associations are not so obvious. For example, sleep apnea, calcinosis, and inborn errors of metabolism are also part of the cluster that has diabetes, hyperglycemia, and hyperlipidemia (Figure 2). Close examination of this cluster shows three pathophysiological processes underway. Diabetes is well known to lead to increased atherosclerosis. Inborn errors of metabolism can lead to impaired energy metabolism and storage. Sleep apnea has been associated with increased oxidative-stress and pulmonary hypertension. But all three seemingly separate processes lead to cardiovascular sequelea such as cerebrovascular accident and myocardial infarction [6-9]. The similarity of these etiologies in leading to nervous system sequelae and postoperative complications also contributed to the clustering. This result suggests that patients with both sleep apnea and diabetes mellitus may be at higher risk for cardiovascular mortality and morbidity. Even though the epidemiology of sleep apnea and diabetes interact [10], studies have shown that the cardiovascular morbidity and mortality remain in patient with sleep apnea even after adjusting for diabetes [9].

We also examined the location of the genetic factors in the "etiome", focusing on those five genes that were associated with the 863 diseases with the highest article counts: *TP53*, *CTL4*, *TNF*, *ACE*, and *NOS3*. While these five genes were scattered across our clustering of the "etiome", surprisingly, they fell into three distinct areas of the "etiome": oncogenesis, inflammation and immunity, and metabolism.

**Figure 3:**
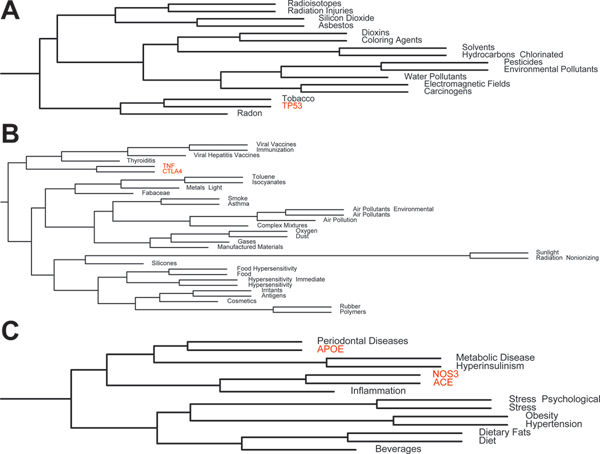
**Sample clustering of genetic and environmental factors**. Branches indicated in Figure 1 are shown in more detail here. **A**. Variants in *TP53 *lead to a set of diseases closely similar to the diseases caused by radon, tobacco, as well as other carcinogens and pollutants. **B**. *CTLA4 *(cytotoxic T-lymphocyte antigen 4) and *TNF *were clustered with immune-mediated processes such as thyroiditis, viral hepatitis vaccines, and immunizations, and more distant similarity with a myriad of environmental pollutants. **C**. Variants in *APOE *lead to the same diseases as periodontal disease, while *ACE *and *NOS3 *share profiles with inflammation. These three genes cluster with metabolic diseases and hyperinsulinism, as etiological factors for other diseases.

Not surprisingly, *TP53 *was clustered with various other carcinogens such as radon, tobacco, electromagnetic fields, pesticides, environmental pollutants, and chlorinated hydrocarbons (Figure 3A). This reflects the role of *TP53 *as "the guardian of the genome" in the face of carcinogens, and specifically reflects the morbidity caused from *TP53 *variants or loss of functioning of this important tumor-suppressor gene.

In another region of the clustered "etiome", *CTLA4 *(cytotoxic T-lymphocyte antigen 4) and *TNF *were clustered with immune-mediated processes such as thyroiditis, viral hepatitis vaccines, and immunizations, and more distant similarity with a myriad of environmental pollutants, such as toluenes, air pollution, polymers, and even silicones (Figure 3B). These two genes share associations with 15 different diseases, including rheumatoid arthritis, asthma, celiac disease, ulcerative colitis, Crohn's Disease, Graves Disease, and multiple sclerosis.

*ACE *and *NOS3 *are associated with inflammation, hyperinsulinism, and metabolic diseases (Figure 3C). *ACE *and *NOS3 *are best known for their role in blood pressure regulation, but both are associated with 28 different diseases, including Alzheimer Disease, unstable angina, brain ischemia, coronary disease, systemic lupus erythematosus, preeclampsia, and autosomal dominant polycystic kidney disease. *ACE *has recently also been implicated in glucose control [11]. Directly neighboring this pair of genes is *APOE*, which is interestingly partnered with periodontal disease. While much has been written about the role of APOE in Alzheimer Disease, the overall profile of APOE across diseases places it as being similar to periodontal disease. Both *APOE *and periodontal disease are known etiologies or complicating factors for arteriosclerosis and its manifestations. Curiously, periodontal disease has been suggested as a risk factor for the development of Alzheimer Disease, but only in a single study [12].

## Discussion

We have integrated publicly available gene-disease and environment-disease relations to build a first pass survey of the "etiome", or a comprehensive profile of disease etiologies, across 863 diseases.

In the past, efforts have been focused on identifying and characterizing the genetic influences on diseases. Recently, a "diseasome" was generated using the Online Mendelian Inheritance in Man (OMIM) database [13]. However, no environmental factors were included in that study, and even more concerning, most of the diseases in OMIM are monogenic diseases that follow Mendelian inheritance. Because of this, many commonly occurring complex polygenic diseases or disorders were not included in that study. This is one of the reasons why we used the NIH Genetic Association Database (GAD) as our primary source of genetic associations, instead of OMIM. A second reason we used GAD is that diseases are already represented by MeSH, which facilitated integration of genetic and environmental etiologies for the same diseases.

To our knowledge, our effort is the first towards building an automated system to collect a comprehensive list of all environmental influences on diseases. We identified 3342 factors belonging to 78 semantic types from a thorough search of human-curated publication annotations from MEDLINE. Combined with the genetic influences, our "etiome" can contribute to our understanding towards genetic and environmental contribution to diseases.

In addition, clustering of genetic and environmental factors together based on their disease profile puts genes in their environmental context, and may even suggest gene-environment interactions. This is reflected in our results with *TP53*, *CTLA4*, *TNF*, *ACE *and *NOS3*. This may become another way to explore gene function, as well as to suggest possible environmental interventions for genetic diseases. While we clustered genes and environmental factors based on the similarities in the diseases they are associated with, careful examination of the subtle differences in these profiles may also indicate gene-disease and environmental-disease relations that are "missing" and might make targeted hypotheses for validation.

We acknowledge that our modeling of gene-environment interactions here is simple and serves only as a first step. Several of the genes we find as having disease-profiles similar to environmental etiologies only express their association under specific environmental conditions. Though we are considering genes and environmental factors as separate when clustered, we realize that in the majority of cases, these factors work together to yield a disease. We also acknowledge that the assignment of genetic associations to genes can operate in a biased manner, in that when one disease is associated with a gene, many investigators may then try to associate other related diseases to that gene.

The distinction between genetic and environmental factors is somewhat arbitrary. We define environmental etiological factors as non-genetic factors that have been associated with the disease from an "etiological" point of view in MEDLINE. As a result, diseases that are predisposing factors to other diseases are included. In reality, many of the disease predispositions themselves could be due to genetic factors. For example, polymorphisms in *APOA4 *have been associated with the risk of myocardial infarction in patients with obesity and type 2 diabetes [14]. These ternary and quaternary relations (i.e. one gene, *APOA4*, and two conditions, obesity and type 2 diabetes, leading to another condition, myocardial infarction) are highly interesting to study, but we acknowledge will be missed by our current approach.

We acknowledge several limitations in the use of MeSH to represent etiological factors. There is variable depth of coverage of scientific areas in MeSH, and while NLM curators may attempt to use the most specific terms for annotate a paper, we acknowledge there may be variable depth of term usage. There may also be a lack of discrimination between environmental factors at different times in the etiologic cascade. This is reflected by the trivial "is a" pairings seen in our clustering. MeSH itself is a dynamically changing structure, with newer terms introduced yearly. Another source of our "is a" pairings might be that certain terms may not have been available for annotation for older publications, and thus these publications may not have been identified with the most specific term currently available.

Our list is nearly comprehensive, but not technically complete. We acknowledge that this first step does not include microorganisms that cause infectious diseases. This is because the MeSH qualifier *microbiology *"permits its use with disease headings for discussions of microbes in a disease whether the microbe is the causative agent or not."  The same is true for *virology *and *parasitology*. To be conservative, we decided not to include these qualifiers in our study to avoid false positive relationships. Infectious diseases and their causative organisms are relatively well characterized. In the future, we plan to look for other sources of information to better delineate the association between infectious agents and the diseases they cause. In addition, we acknowledge that while the NIH Genetic Association Database is human curated, it may be missing gene-disease relationships.

We also acknowledge that we cannot distinguish positive and negative findings solely by using MeSH annotations. For example, a publication showing that the use of hormone replacement therapy has no effect on breast cancer incidence has the same MeSH annotations as a publication showing that hormone replacement therapy is statistically associated with increasing breast cancer incidence. Distinguishing positive from negative findings will likely require natural language processing of the title and abstracts of these publications, or using other curated sources such as the Cochrane collection. Our criteria for inclusion in this study is minimally filtered, in that if any publication exists linking etiology with disease, we consider that link as a positive association. Given the known biases in publication acceptance, it is probably rare that a negative finding is published without a preceding positive and opposite finding, so we feel that potential inclusion of these negative studies is justified given that there was likely a positive study to precede them. However, this assumption is unverified.

We acknowledge that we are missing actual odds ratio, relative risk or penetrance of these etiological factors and their associated disease. To our knowledge, we are unaware of any repositories with this type of quantifiable data, but we also submit that any quantifiable odds ratios are likely to be a factor of the experimental design and conditions of a particular study, which are currently ignored in our work.

Our future work will include refining our collection of etiological factors, clustering the diseases as reflected by our "etiome", and building graphical networks between genes, environmental factors, and diseases. The clustering of iatrogenic and health-care related sources of disease with genetic factors is of particular interest to us, as this suggests that two disparate fields of research, quality of care improvement and genetic associations, could be used together to jointly model the incidence of disease. This is of prime importance given the current high estimates for the role of medical errors in mortality and morbidity.

In addition, most of our primary sources of relations have an implicit temporality, in terms of when each relation was discovered or published. We envision retrospective time-series studies of changes to determine how our dynamic view of the role of genetics and environment on disease can be demonstrated through changes in the clustering of the "etiome."

## Conclusion

In this paper, we obtained a comprehensive set of associations between disease and etiological factors from the NIH Genetic Association Database and MeSH annotations of MEDLINE articles. We computed the profiles across the "etiome" for 863 diseases, and clustered these etiological factors. This is just a first-step towards the automated analysis of gene-environmental relationships across diseases. Our work may have value for both clinical medicine and basic science research: clustering of environmental factors may alert clinicians of the risks of added exposure and clustering of genetic and environmental etiological factors may reveal novel gene functions and dysfunctions.

## Methods

### Identifying disease-gene associations

The Genetic Association Database (GAD) is a curated archive of human genetic association studies supported by the US National Institutes of Health [5]. The October 2006 version of GAD was downloaded from . Only positive genetic associations were selected; relations indicating negative findings were removed. Relations in GAD link NCBI Entrez Gene identifiers to diseases as represented by MeSH terms. Each separate article noting a relationship between a gene and disease is initially counted separately, so that article counts are maintained.

### Clustering of etiological factors

Associations between diseases and genes, or diseases and environmental factors are represented as binary values in a table with diseases as rows and gene/environmental factors as columns. Hierarchical clustering is performed on etiological factors using binary distance and complete agglomeration method. Computations were performed using R [15]. In order for the dendrogram to be easily visualized, only those etiological factors associated with the highest article counts are clustered.

### Availability of data

Data and magnified figures can be downloaded from .

## Competing interests

The authors declare that they have no competing interests.

## Authors' contributions

All authors participated in the design of the study. YIL carried out the acquisition of data. YIL and AJB analyzed the data. All authors read and approved the final manuscript.
